# 

**Published:** 1921-02-19

**Authors:** 


**The Modern Newspaper of Administrative Medicine and Institutional Life.**

Telegraphic Address: OSPEDALE, RAND, LONDON. Telephone Number: 2734 GERRARD.

No. 1811, Vol. LXIX. | Saturday, February 19th, 1921. | PRICE TWOPENCE.

